# Complete mitochondrial genome sequencing of *Lutra lutra* (Linnaeus, 1758) (Carnivora: Mustelidae) and its phylogenetic status in Mustelidae

**DOI:** 10.1080/23802359.2021.1942274

**Published:** 2021-06-21

**Authors:** Han-Na Kim, Yeong-Seok Jo

**Affiliations:** Biology Education, Daegu University, Gyeongsan, South Korea

**Keywords:** *Lutra lutra*, Lutrinae, mitochondrial genome, Mustelidae

## Abstract

We report the complete mitochondrial genome sequence of the endangered Eurasian otter, *Lutra lutra*. The complete mitochondrial genome is 16,537 bp in length and contains 13 protein-coding genes, 22 transfer RNA, two ribosomal RNA, and one control region. The mitogenome is A + T rich, with a composition of 32.2% A, 27.5% C, 14.5% G, and 25.8% T. Phylogenetic analysis based on 13 protein-coding mitochondrial genes of Mustelidae supports the conventional systematic treatment with eight subfamilies. *Lutra lutra* is closely related to *Lutra sumatrana* and the subfamily Lutrinae was closely grouped with the Ictonychinae. This study provides genetic and taxonomic information for future studies of Eurasian otters and the Mustelidae.

The Eurasian otter, *Lutra lutra* (Linnaeus, 1758) (Carnivora, Mustelidae, and Lutrinae) is a cosmopolitan species widely distributed from Western Europe to north-eastern Siberia and the Korean Peninsula (Jo et al. 2018). The status of Eurasian otters has been regarded as “near threatened” by the Red List of the International Union for Conservation of Nature (IUCN). Also, the Conservation on International Trade in Endangered Species (CITES) has listed the species in Appendix I (Roos et al. 2015). European populations of *Lutra lutra* have been declining and some local populations have been extirpated (Ferrari et al. 2017). Despite the global decline and social interests, only a limited number of genetic studies on the Eurasian otter have been published (Jo et al. 2012, 2017). In particular, few genomic studies have been performed (NIBR 2019).

Currently, the Mustelidae including the otter subfamily, Lutrinae, is subdivided into eight subfamilies based on the limited number of genetic markers and morpho-anatomical studies of fossils (Koepfli et al. 2008; Law et al. 2018). Unfortunately, the limited numbers of genetic markers or small marker lengths can sometimes fail to fully resolve the true evolutionary relationships among populations (Koepfli and Wayne 2003; Yu et al. 2008). To clarify the phylogenetic analysis of the subfamily and family, more molecular markers are required (Morton and Telmer 2014).

Here we sequenced the complete mitochondrial genome for *Lutra lutra* and analyzed the phylogenetic history of members classified to the subfamilies of Mustelidae. The voucher specimen was a road-killed Eurasian otter from the Daejeon metropolitan area in South Korea (36°21′35.13″N 127°22′46.22″E), and the study skin and skull with extracted DNA are stored at the Daegu University in South Korea (Voucher No. DUMM0035, the animal specimen storage room in the department of biology education [https://bioedu.daegu.ac.kr/] curator: Prof. Y. S. Jo [fright@daegu.ac.kr]). DNA was extracted from muscle tissue using a QIAGEN blood and tissue kit (Qiagen, Valencia, CA, USA), and genomic DNA was sequenced using the HiseqX platform (Illumina, San Diego, CA, USA). The assembly was performed *de novo* using the low coverage whole genome shotgun sequencing (dnaLCW) method from PHYGEN (Seongnam, Korea). We employed the GeSeq program (https://chlorobox.mpimp-golm.mpg.de/geseq-app.html, Tillich et al. 2017) for the annotation.

The complete mitochondrial genome of *L. lutra* is 16,537 bp in length (MW573979) and contains 13 protein-coding genes (ND1-ND6; COXI-3; ATP6 and ATP8; CYT B), 22 transfer RNA, two ribosomal RNA, and one control region (D-loop). The base composition of the mitochondrial genome of Eurasian otters from Korea is 32.2% A, 27.5% C, 14.5% G, and 25.8% T with the total A-T content (58%) higher than G–C content (42%). The 12S rRNA and 16 s rRNA are 964 and 1568 bp, respectively. The 12S rRNA gene is located between RNA^Phe^ and tRNA^Val.^ and the 16S rRNA gene is positioned between tRNA^Val^ and tRNA^Leu^. The ND and eight tRNAs are encoded in the reverse strand, while the remainder is on the forward strand. There are 10 PCGs initiate with the ATG as a start codon, while ND2 initiates with ATC, and ND3 and ND5 begin with ATA. Incomplete stop codons are found in ND2 and COX3 (T-), and ND1 (TA-). For Cytb, AGA terminates the gene and the other nine genes terminate with TAA.

For the phylogenetic analysis, 13 protein-coding genes (PCGs) representing eight subfamilies were downloaded from GenBank, including five genera of Lutrinae. As the outgroup, the Leopard cat, *Prionailurus bengalensis* (Kerr, 1792) (Carnivora, Felidae) (KP246843) was designated. Seventy-one individuals mitochondrial PCG sequences from 39 species were aligned with MAFFT v.7040 using default settings (Katoh et al. 2002; Katoh and Standley 2013). We employed RAxML v.8.2.11 (Stamatakis 2014) for reconstructing the phylogenetic tree with 1000 bootstrap replicates and the default general time-reversible GTR + GAMMA model. The model supports the conventional eight monophyletic subfamilies in Mustelidae (Figure 1). The Korean population of the otter forms a sister group to *Lutra sumatrana* (bootstrap value, 100). The Asian small-clawed otter, *Aonyx cinerea*, was grouped with the smooth-coated otter, *Lutrigale perspicillata*, rather than the Asian clawless otter, *Aonyx capenis*. Although the previous studies reported that the Mustelinae is the closest subfamily of the Lutrinae (Moretti et al. 2017), these results show that the Ictonychinae and Lutrinae were unresolved in a clade separate from the Mustelinae, Helictidinae, and Guloinae. These results based on mitochondrial genomic markers generally coincided with the previous studies (Koepfli et al. 2008; Law et al. 2018). The Korean *L. lutra* from Daejeon differed from *L. lutra* from other specimens from Korea, GenBank accession number NC_011358 (exact location is unknown, expect Korea; Jang et al. 2009 ) by 25 SNPs and one indel (9 bp in the length), from GenBank accession number EF672696 (exact location is unknown, expect Korea; Ki et al. 2010) by 29 SNPs and two indels (12 and 15 bp in the length). The genomic resources of the Korean *L. lutra* obtained from this study provide an additional reference for genetic studies as well as conservation and management for the Eurasian otters.

**Figure 1.**
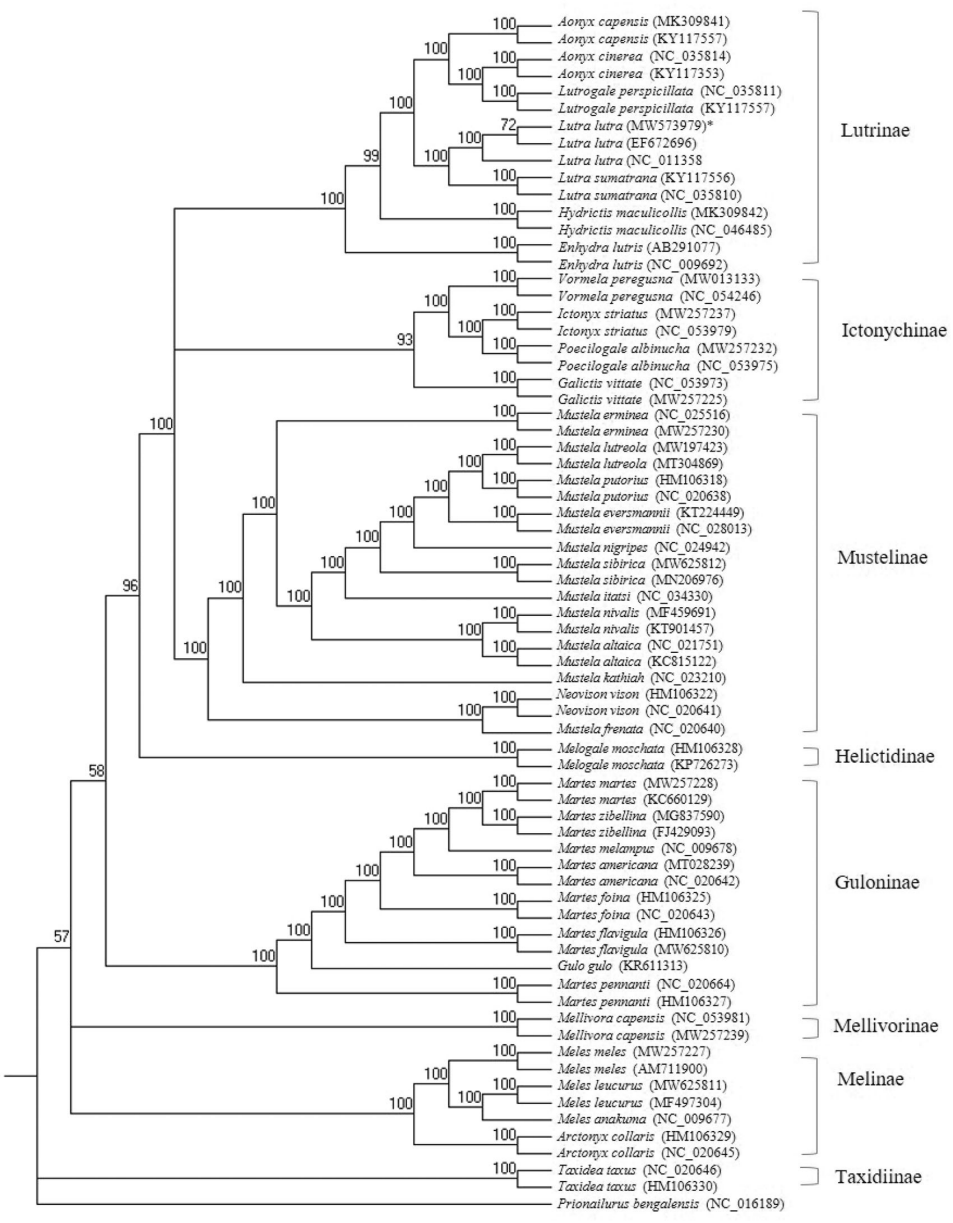
The phylogenetic relationship of the *Lutra luta* and other Mustelidae species based on 13 PCGs sequences using RAxML program with *Prionailurus bengalensis* as an outgroup. The numbers on the branches indicate bootstrap value. *Indicates the Korean otter *Lutra lutra* used in this study with the mitogenome accession no. MW573979.

## Data Availability

The genome sequence data that support the findings of this study are openly available in GenBank (National Center for Biotechnology Information) at https://www.ncbi.nlm.nih.gov, accession no. MW573979. The associated BioProject, SRA, and BioSample numbers are PRJNA725629, SRR14478347, and SAMN18897176, respectively. The data that support the findings of this study are also openly available in Mendeley Data at http://dx.doi.org/10.17632/74bpx3kfx3.1.
